# Essential Oil Composition and Antimicrobial Activities of Two Closely Related Species, *Alpinia mutica* Roxb. and *Alpinia latilabris* Ridl., from Peninsular Malaysia

**DOI:** 10.1155/2014/430831

**Published:** 2014-05-27

**Authors:** Halijah Ibrahim, Yasodha Sivasothy, Devi Rosmy Syamsir, Noor Hasima Nagoor, Natasha Jamil, Khalijah Awang

**Affiliations:** ^1^Institute of Biological Sciences, Faculty of Science, University of Malaya, 50603 Kuala Lumpur, Malaysia; ^2^Department of Chemistry, Faculty of Science, University of Malaya, 50603 Kuala Lumpur, Malaysia

## Abstract

The essential oils obtained by hydrodistillation of the unripe and ripe fruits of *Alpinia mutica* Roxb. and *Alpinia latilabris* Ridl. were analysed by capillary GC and GC-MS. The oils were principally monoterpenic in nature. The unripe and ripe fruit oils of *A. mutica* were characterized by camphor (21.0% and 15.8%), camphene (16.6% and 10.2%), **β**-pinene (8.6% and 13.5%), and *trans,trans*-farnesol (8.0% and 11.2%), respectively. The oils of the unripe and ripe fruits were moderately active against *Staphylococcus aureus, Bacillus subtilis, Trichophyton mentagrophytes,* and *Trichophyton rubrum*. 1,8-Cineole (34.2% and 35.9%) and **β**-pinene (20.2% and 19.0%) were the two most abundant components in the unripe and ripe fruit oils of *A. latilabris*. The oil of the unripe fruits elicits moderate activity against *Staphylococcus aureus* and *Trichophyton mentagrophytes* while *Candida glabrata* was moderately sensitive to the oil of the ripe fruits.

## 1. Introduction

*Alpinia* is one of the largest genera in the Zingiberaceae and it is widely distributed in the Southeast Asian region. In Malaysia and Indonesia, the rhizomes of the common* Alpinia* species,* A. galanga *Willd. known as* lengkuas*, are mainly used to flavour food. In Malay traditional use, the leaves of this plant are boiled and used as a body lotion [1].* A. conchigera* Griff., locally known as* lengkuas ranting* and* lengkuas kecil*, is used for treating rheumatism after child birth [1]. Several other species of* Alpinia* are used as ornamentals due to the beautiful inflorescence and flowers such as* A. purpurata* K. Schum.,* A. zerumbet* (Pers.) Burt and R. M. Smith, and* A. mutica*. It is interesting to investigate the chemistry and the bioactivity of the* Alpinia* species in particular, the wild species which are yet to be exploited. Some of the essential oils of the* Alpinia* species were reported to exhibit various bioactivities such as antimicrobial, larvicidal, and antioxidant  [2–5].

*A. mutica* is a herbaceous perennial plant, 1.2–1.7 m tall, indigenous to Peninsular Malaysia and Thailand. Several variants can be observed in the wild, but the type species are distributed within the northern part of Peninsular Malaysia where the specimen used in this study was collected from. The leaves are distichous and narrowly elliptic. The fruits are spherical, less than 2 cm in diameter, and sparsely covered with hairs and the edible fruits turn orange when ripe. This plant is commonly used by the locals to treat flatulence and the fruits are used by some village folk to treat diarrhoea (pers. comm). In comparison, * A. latilabris* is a taller plant with broader leaves that grows up to 3 m or more and found wild in Peninsular Malaysia [6]. The flowers are yellow with crimson spots and stripes, orchid-like, and borne in an inflorescence. The fruits are more or less globose, 2–2.5 cm in diameter, and covered with many stiff hairs. The unripe fruits are green turning orange on ripening. The ripe fruits contain many aromatic seeds and are edible. Both the rhizomatous and perennial species are naturally grouped together within the subsection Catimbium [7] as their floral characteristics are closely similar to one another. * A. mutica* has been frequently planted in gardens and public parks; however, due to rapid development and deforestation activities, both species are becoming rare in the wild.

In our continuous effort to study the essential oils of the Zingiberaceae species and their antimicrobial activities [2, 3, 8, 9], the present study aims to investigate the essential oil composition of the unripe and ripe fruit oils of* A. mutica* and* A. latilabris* and their antimicrobial activity.

## 2. Materials and Methods

### 2.1. Plant Material and Reagents

The unripe and ripe fruits of* A. mutica* and* A. latilabris*, authenticated by Professor Halijah Ibrahim, were collected from the Rimba Ilmu Botanic Garden, in the vicinity of University Malaya in October 2009. Voucher specimens (HI 1417 and HI 1418, resp.) have been deposited in the university herbarium. Pentane (GC-MS grade) and the homologous series of n-alkanes (C_6_–C_30_) were purchased from Merck (Germany) and Dr. Ehrenstorfer Gmbh (Germany), respectively.

### 2.2. Isolation of Essential Oils

Fresh unripe and ripe fruits of* A. mutica* (280 g and 38 g) and* A. latilabris* (1200 g and 290 g), respectively, were separately hydrodistilled for 4 hours in an all-glass apparatus similar to that described in the British Pharmacopoeia using pentane as the collecting solvent [10]. The solvent was carefully removed using a gentle stream of nitrogen gas, yielding aromatic oils in each case. The oil yields (w/w) were* A. mutica*: 0.04% (unripe fruits) and 0.16% (ripe fruits);* A. latilabris*: 0.08% (unripe fruits) and 0.05% (ripe fruits), all on a fresh weight-basis.

### 2.3. Gas Chromatography (GC) Analysis

GC analysis was carried out using an Agilent 7890A GC System equipped with a FID and an Agilent 7683B Series autoinjector. A HP-5MS UI (30 m × 0.25 mm id, film thickness 0.25 *μ*m) fused-silica capillary column was employed. Operating conditions were as follows: initial oven temperature, 60°C for 10 mins, then to 230°C at 3°C min^−1^ and held for 20 mins; injector and detector temperatures, 250°C; carrier gas, 1.0 mL min^−1^ N_2_; injection volume, 0.2 *μ*L; split ratio, 20 : 1. Quantitative data were obtained electronically from FID area percent without the use of correction factors.

### 2.4. Gas Chromatography-Mass Spectrometry (GC-MS) Analysis

GC-MS analysis was performed using an Agilent 6890N Network GC System equipped with an Agilent 7683 Series autoinjector coupled to an Agilent 5975 Inert Mass Selective Detector and the same capillary GC conditions as described above. Carrier gas used was He at a flow rate of 1.0 mL min^−1^. Significant MS operating parameters were ionization voltage, 70 eV;  ion source temperature, 230°C;  and mass range, 50–600 u.

### 2.5. Identification of Constituents

Constituents were identified by comparison of their mass spectra with those of authentic compounds or with reference spectra in the computer library (NIST 05) and confirmed by comparison of retention indices with those of authentic compounds or with data in the literature [8, 9, 11].

### 2.6. Antimicrobial Assay

#### 2.6.1. Test Microorganisms

The essential oils were tested against a panel of eight microorganisms:* Staphylococcus aureus *(ATCC 25923), a methicillin susceptible* S. aureus* isolate*, Bacillus subtilis* (ATCC 6633),* Escherichia coli* (ATCC 8739),* Pseudomonas aeruginosa* (ATCC 27853),* Candida glabrata* (ATCC 64677),* Microsporum canis* (ATCC 36299),* Trichophyton mentagrophytes* (ATCC 18748), and* Trichophyton rubrum* (ATCC 28188). The inoculum was adjusted to obtain a turbidity comparable to that of McFarland standard tube number 0.5 [12] for further use.

#### 2.6.2. Minimum Inhibitory Concentration (MIC)

Media were sterilized by autoclaving at 120°C for 15 minutes and all subsequent manipulations were carried out in a class 2 laminar flow cabinet. The effectiveness of the antifungal and antibacterial activities of the essential oils was quantified in liquid media by employing the microdilution method using microtiter plates (12 × 8 wells). 10 *μ*L of the stock solution (50 mg/mL) of each essential oil in dimethyl sulfoxide (DMSO) (not more than 10% of total volume in well A) and 90 *μ*L of broth were added to the well labeled as A. Only 50 *μ*L of broth was added to wells labeled as B until H. The oils and broth in well A were mixed thoroughly before transferring 50 *μ*L of the resultant mixture into well B. The same procedure was repeated for mixtures in well B until H, thus creating a serial dilution of the test materials. 50 *μ*L of inoculum (microbes tested) was added to well A to well H. The microtiter plates were then incubated at 37°C for 24 hours. Cycloheximide (50 mg/mL) was used as the standard antibiotic for comparison with the antifungal activities of the essential oils while Oxacillin (50 mg/mL) was used as the standard for the antibacterial testing. DMSO served as the negative control. Turbidity was taken as indication of growth; thus, the lowest concentration which remains clear after macroscopic evaluation was taken as the minimum inhibitory concentration (MIC). The MIC was recorded as a mean concentration of triplicates. The activities were categorized as weak (MIC ≥ 5.0 mg/mL), moderate (MIC 1 mg/mL–4.9 mg/mL), and strong (MIC ≤ 1 mg/mL).

## 3. Results and Discussion

Table 1 lists the constituents identified in the essential oils of the unripe and ripe fruits of * A. mutica* and * A. latilabris*, the relative GC peak areas of these constituents, and their experimental retention indices on the HP-5 MS UI column.

53 constituents were identified in the unripe fruit oil of* A. mutica* while the ripe fruit oil yielded 60 identified constituents. Monoterpenoids dominated the volatile profile of the unripe and ripe fruit oils, contributing to total 74.0% and 71.9%, respectively. These figures were largely due to camphor (21.0% and 15.8%), camphene (16.6% and 10.2%), **β**-pinene (8.6% and 13.5%), 1,8-cineole (5.1% and 9.6%), and **α**-pinene (5.7% and 6.9%), respectively, the first being the most abundant component in both oils. Sesquiterpenoids were significant in number, totaling 18.4% in the unripe fruit oil and 21.1% in the oil of the ripe fruits, with* trans,trans*-farnesol clearly predominating (8.0% and 11.2%, resp.). Comparison of the composition of the unripe and ripe fruit oils of * A. mutica* with those previously examined by Sirat and coworkers revealed marked differences [13]. In contrast to the present analysis, the oils reported by Sirat and her coworkers identified high yields of sesquiterpenoids (74.4%–77.4%). Among the 44 and 41 constituents identified in the young and matured fruits, respectively, by the previous group, 31 were found to be common in the present investigation [13]. With regard to camphor, camphene, **β**-pinene, and **α**-pinene, the compounds which characterized the fruit oils in this study, they were only detected at concentrations below 2.0% in the previous investigation [13].* trans,trans*-Farnesol, the most abundant constituent in the fruit oils isolated by Sirat et al. (44.3%–51.2%), only made up a smaller fraction of the sesquiterpenoid composition in the present investigation [13]. Unlike Sirat et al., the exact isomers for **β**-farnesene, **α**-farnesene, and **α**-bergamotene were determined in this analysis. However, we were not able to detect 3-phenyl-2-butanone, carvacrol, **α**-cubebene, **β**-elemene, **γ**-elemene, **β**-bisabolene, docosane, tricosane, tetracosane, and pentacosane. These differences may have been attributed to the source, cultivation, vegetative stage, and the growing season of the species under investigation [14].

Analysis of the unripe and ripe fruit oils of* A. latilabris* resulted in the identification of 45 and 44 constituents, respectively. In contrast to its rhizome oil being rich in phenylpropanoids, in particular* trans*-methyl cinnamate (89.5%) [15], monoterpenoids (91.7% and 92.1%) characterized the oils of the unripe and ripe fruits, respectively, with 1,8-cineole (34.2% and 35.9%), **β**-pinene (20.2% and 19.0%), **α**-pinene (8.2% and 8.8%), camphor (7.4% and 8.8%), and camphene (5.1% and 5.8%) accounting for more than half of each sample, respectively.

Overall, the similarity of compounds in the unripe and ripe fruits of* A. mutica* and* A. latilabris* is 48% and 61%, respectively. Botanically,* A. mutica* and* A. latilabris* are believed to be two closely related species. Preliminary molecular study of the length and GC (guanine, cytosine) composition of internal transcribed spacer (ITS1 and ITS2) regions of these two species showed that both species have the same length for ITS1 (177 bp) and ITS2 (224 bp) spacer. The total GC content of* A. latilabris *(55.1% in ITS1 and 58.9% in ITS2) varies only slightly from that of* A. mutica* (54.7% in ITS1 and 57.6 in ITS2). The relatively high similarities in the chemical composition and the preliminary molecular data implicate that these two species are closely related.

The unripe and ripe fruit oils of* A. mutica* and* A. latilabris* were tested against two Gram-positive bacteria (*Staphylococcus aureus* and* Bacillus subtilis*), two Gram-negative bacteria (*Escherichia coli* and* Pseudomonas aeruginosa*), and four fungal strains (*Candida glabrata*,* Microsporum canis*,* Trichophyton mentagrophytes*, and* Trichophyton rubrum*) as showed in Table 2. Both the unripe fruit oil and ripe fruit oil of* A. mutica* showed antibacterial activity against* B. subtilis* (2.50 mg/mL and 1.25 mg/mL, resp.) and* S. aureus* (2.50 mg/mL for both). The ripe fruit oil showed the highest activity towards* B. subtilis*; however, it is about ninefold less active than the standard, oxacillin. Both oils also showed potency of 2.50 mg/mL to 5.0 mg/mL against the dermatophytes* M. canis, T. mentagrophytes,* and* T. rubrum*. Interestingly both oils exhibited the same potency as the standard, cycloheximide against* T. mentagrophytes*. Overall the ripe fruit oil of* Alpinia mutica* exhibited slightly higher activity (lower MIC against* B. subtilis* and* M. canis*) as compared to the unripe fruit. Both oils, however, showed no activity (MIC > 5.0 mg/mL) against the pathogens* E. coli, P. aeruginosa, *and* C. glabrata*.

The oils of* A. latilabris*, both unripe and ripe, showed inhibition towards the bacteria* S. aureus* and* B. subtilis* with MIC values between 2.50 mg/mL and 5.0 mg/mL. However, only the ripe fruit oil showed weak activity against* E. coli* and* P. aeruginosa* with MIC value of 5.0 mg/mL. Both oils also elicited antifungal activities against the dermatophytes* M. canis, T. mentagrophytes,* and* T. rubrum* with MIC values of 2.50 mg/mL to 5.0 mg/mL. On the other hand, only the ripe fruit oil showed moderate potency against* C. glabrata* while the unripe oil is void of activity. Interestingly, the unripe fruit oil of* A. latilabris* showed the same MIC value against* T. mentagrophytes* as that of cycloheximide. Therefore, both the unripe and ripe oils of* A. mutica* and the unripe fruit oil of* A. latilabris* showed similar antifungal activity as the antifungal drug, cycloheximide.

## 4. Conclusions

The unripe and ripe fruit oils of both* A. mutica* and* A. latilabris* were principally monoterpenic in nature. The unripe and ripe fruit oils of* A. mutica* were characterized by high levels of camphor (21.0% and 15.8%) and camphene (16.6% and 10.2%) while those of* A. latilabris* were dominated by 1,8-cineole (34.2% and 35.9%) and **β**-pinene (20.2% and 19.0%). In general, the fruit oils exhibited weak to moderate antimicrobial activity. However, it is interesting to note that both unripe and ripe fruit oils of* A. mutica* and the unripe fruit oil of* A. latilabris *showed similar potency as the standard drug, cycloheximide, against* Trichophyton mentagrophytes*.

## Figures and Tables

**Table 1:** Essential oil constituents of the flowers, unripe and ripe fruits of *A. mutica* Roxb. and *A. latilabris* Ridl.

Constituent	RI (HP-5MS UI)	Area (%)^a^			
		*A. mutica *		*A. latilabris *	
		Unripe fruits	Ripe fruits	Unripe fruits	Ripe fruits
2-Heptanone	888	*t*	0.1	—	—
2-Heptanol	899	0.7	0.5	0.8	0.7
Tricyclene	920	0.4	0.3	0.1	0.2
*α*-Thujene	925	0.1	0.1	—	0.2
*α*-Pinene^b^	935	5.7	6.9	8.2	8.8
Camphene^b^	948	16.6	10.2	5.1	5.8
Benzaldehyde	959	0.1	0.1	—	0.1
Sabinene	972	—	0.1	—	—
*β*-Pinene^b^	980	8.6	13.5	20.2	19.0
6-Methyl-5-hepten-2-one	987	*t*	0.1	—	0.1
Myrcene^b^	991	3.2	2.1	2.1	1.6
*α*-Phellandrene^b^	1003	1.0	1.2	2.5	0.7
*α*-Terpinene	1017	0.1	0.1	—	—
*p*-Cymene^b^	1024	0.3	0.5	—	0.1
Limonene	1029	4.2	2.9	0.2	—
1,8-Cineole^b^	1031	5.1	9.6	34.2	35.9
*cis*-*β*-Ocimene^b^	1037	*t*	*t*	—	—
2-Heptyl acetate	1048	0.1	—	*t*	—
*trans*-*β*-Ocimene^b^	1050	0.1	0.1	—	0.4
*γ*-Terpinene^b^	1059	*t*	0.2	0.8	0.3
Octanol	1071	0.1	—	—	—
*trans*-Linalool oxide (furanoid)	1073	—	*t*	0.1	—
Fenchone	1083	—	*t*	—	—
Terpinolene^b^	1088	0.4	0.3	0.3	0.3
2-Nonanone	1090	0.3	0.2	0.1	0.1
Linalool^b^	1099	1.8	1.5	2.7	2.3
Fenchol^b^	1115	*t*	0.1	—	*t*
*trans*-Sabinene hydrate	1117	—	0.1	—	—
*cis*-*p*-Menth-2-en-1-ol	1121	—	0.1	—	—
Camphor^b^	1143	21.0	15.8	7.4	8.8
Camphene hydrate	1147	—	—	0.3	—
*β*-Pinene oxide	1162	—	0.1	0.1	—
Isoborneol^b^	1163	0.5	0.4	0.3	0.3
Borneol^b^	1168	0.9	0.8	1.8	2.2
Terpinen-4-ol^b^	1176	0.6	0.5	1.0	1.0
*p*-Cymen-8-ol	1184	0.1	—	—	—
*α*-Terpineol^b^	1190	2.2	1.9	3.2	3.1
Myrtenal^b^	1196	0.1	0.1	—	0.3
Myrtenol	1197	—	—	0.1	0.1
*trans*-Piperitol	1208	*t*	—	—	—
*β*-Citronellol^b^	1228	0.1	0.6	*t*	0.1
Neral	1238	—	*t*	0.1	0.1
Geraniol^b^	1255	0.3	1.0	0.3	—
Geranial	1270	—	0.5	0.1	0.2
Bornyl acetate^b^	1289	0.4	0.2	0.4	0.2
Thymol	1294	—	*t*		
2-Undecanone	1299	*t*	0.1	0.1	—
Carvacrol	1299	—	—	—	0.1
Methyl geranate	1324	—	*t*	—	—
*α*-Cubebene	1350	—	—	*t*	0.1
Neryl acetate	1367	*t*	—	—	—
*α*-Copaene^b^	1372	0.1	—	—	—
Geranyl acetate^b^	1380	0.2	0.1	0.1	—
*β*-Elemene	1402	—	—	*t*	—
*β*-Caryophyllene^b^	1419	2.5	2.1	1.0	0.5
*trans*-*α*-Bergamotene	1435	0.4	0.4	—	0.2
*α*-Humulene^b^	1454	0.3	0.2	0.1	0.1
*trans*-*β*-Farnesene	1456	0.1	0.1	—	0.1
*allo*-Aromadendrene	1457	—	—	0.1	—
*β*-Santalene	1461	—	*t*	—	—
*ar*-Curcumene^b^	1481	0.2	0.2	0.2	0.2
*α*-Zingiberene^b^	1495	0.4	0.3	0.1	0.1
*trans,trans*-*α*-farnesene	1507	1.9	1.9	0.5	0.4
*γ*-Cadinene	1515	—	0.2	—	—
*β*-Sesquiphellandrene	1523	1.4	1.0	0.5	0.4
*α*-Elemol	1534	—	—	0.1	—
Germacrene B	1561	—	*t*	—	—
*trans*-Nerolidol^b^	1563	0.9	1.2	1.1	1.0
Caryophyllene oxide^b^	1583	0.8	1.3	—	0.6
*γ*-Eudesmol	1631	—	—	0.1	—
*β*-Eudesmol	1644	—	—	0.1	—
*α*-Cadinol	1653	0.1	—	0.2	—
*α*-Santalol	1680	0.4	0.4	—	—
*α-*Bisabolol	1684	0.9	0.3	—	0.2
*cis,cis*-Farnesol	1717	—	—	—	0.5
*trans,trans*-Farnesol^b^	1723	8.0	11.2	—	—
*trans,trans*-Farnesal	1739	—	0.3	—	0.2
*trans,cis*-Farnesol	1743	—	—	0.2	—

		93.7%	94.1%	97.0%	97.7%

^a^Percentage of total FID area obtained on HP-5 MS UI column, *t* = (<0.05%).

^b^Previously reported in the young and matured fruits of *A. mutica* Roxb. by Sirat et al., 2009 [13].

**Table 2:** Antimicrobial activity (MIC) of the unripe and ripe fruit oils of *A. mutica* and *A. latilabris. *

Microorganisms	MIC (mg/mL)*					
	*A. mutica *		*A. latilabris *		Standards	
	Unripe fruit oil	Ripe fruit oil	Unripe fruit oil	Ripe fruit oil	Oxacillin	Cycloheximide
*Staphylococcus aureus* (ATCC 25923)	2.50	2.50	2.50	5.00	0.20	n.t
*Bacillus subtilis* (ATCC 6633)	2.50	1.25	5.00	5.00	0.14	n.t
*Escherichia coli* (ATCC 8739)	>5.00	>5.00	>5.00	5.00	0.65	n.t
*Pseudomonas aeruginosa* (ATCC 27853)	>5.00	>5.00	>5.00	5.00	5.08	n.t
*Candida glabrata* (ATCC 64677)	>5.00	>5.00	>5.00	2.50	n.t	0.04
*Microsporum canis* (ATCC 36299)	5.00	2.50	5.00	5.00	n.t	1.88
*Trichophyton mentagrophytes* (ATCC 18748)	2.50	2.50	2.50	5.00	n.t	2.50
*Trichophyton rubrum* (ATCC 28188)	2.50	2.50	5.00	5.00	n.t	1.88

n.t = not tested.

*Weak (MIC ≥ 5.0 mg/mL), moderate (MIC 1 mg/mL–4.9 mg/mL), and strong (MIC ≤ 1 mg/mL).
